# A fast privacy-preserving patient record linkage of time series data

**DOI:** 10.1038/s41598-023-29132-8

**Published:** 2023-02-25

**Authors:** Ahmed Soliman, Sanguthevar Rajasekaran, Patrick Toman, Nalini Ravishanker

**Affiliations:** 1 Computer Science and Engineering Department, University of Connecticut, Storrs, CT 06269 USA; 2 Department of Statistics, University of Connecticut, Storrs, CT 06269 USA

**Keywords:** Statistics, Computer science, Public health

## Abstract

Recent advances in technology have led to an explosion of data in virtually all domains of our lives. Modern biomedical devices can acquire a large number of physical readings from patients. Often, these readings are stored in the form of time series data. Such time series data can form the basis for important research to advance healthcare and well being. Due to several considerations including data size, patient privacy, etc., the original, full data may not be available to secondary parties or researchers. Instead, suppose that a subset of the data is made available. A fast and reliable record linkage algorithm enables us to accurately match patient records in the original and subset databases while maintaining privacy. The problem of record linkage when the attributes include time series has not been studied much in the literature. We introduce two main contributions in this paper. First, we propose a novel, very efficient, and scalable record linkage algorithm that is employed on time series data. This algorithm is 400× faster than the previous work. Second, we introduce a privacy preserving framework that enables health institutions to safely release their raw time series records to researchers with bare minimum amount of identifying information.

## Introduction

Recent advances in technology have led to an explosion of data in virtually all domains of our lives. The biomedical or healthcare domain is no exception. Modern biomedical devices can acquire and store a large number of physical readings from patients. Often, these readings are in the form of time series data, such as, for example, heart rates measured once every second for several hours. The devices can range from full-featured ergometers operated by health institutions to wearable devices used on their own by the end users (e.g., Fitbits). Such records are usually saved in huge data bases and form the basis for patient care as well as important and interesting primary research to advance healthcare and well being. Due to several considerations including data size, patient privacy, etc., the original, full data cannot be made available to secondary parties or researchers. Instead, a subset of the data is made available in which personal information has been masked. A fast and reliable *record linkage* algorithm is necessary for accurately matching patient records in the original and subset databases, while maintaining privacy.

Broadly speaking, *record linkage* refers to the set of methodologies and algorithms designed to identify and merge records for the same individual or entity across multiple files or databases^1^. The approach has also been referred to as data matching, entity resolution, or data linkage. Record linkage is ubiquitous in many domains and becomes necessary when it is required to join different data sets based on entities that may or may not share a common identifier/key (such as a social security number). A general version of the methodology thus takes as input several data sets. Each data set contains records pertaining to entities. The same entity may have multiple records in the different data sets. The problem is to identify all the records belonging to each entity, and link them across the data sets. For instance^2^, discuss record linkage with reference to World Trade Center (WTC) registries.

Record linkage is very useful in epidemiological and healthcare applications. In this context, a record typically consists of a collection of attributes corresponding to a patient, such as first name, last name, gender, date of birth, height, etc. For instance, in clinical trials, record linkage helps researchers to leverage the full power of the trials, particularly because these algorithms can enable a scientist to merge information from several clinical trials into one big data set. Analyzing this consolidated data set could then lead to better results.

Indeed, record linkage has found numerous applications in the health sciences. For example, the authors of^3^ give several different examples of the application of administrative record linkage methods with the goal of improving public health research. In addition to public health research, record linkage also has been seen enjoyed widespread utility within the domain of clinical trials. For example, the authors of^4^ employed a record linkage strategy to evaluate treatment outcomes for all cancer patients in the NHS Greater Glasgow and Clyde healthcare system. Another application of record linkage within a clinical setting is given by^5^ in which a novel record linkage is used to ascertain the validity of offspring-reported parental hip fractures using records from the province of Manitoba. Finally, another recent example^6^ describes the approach to record linkage used by CPRD and NHS Digital, a statutory body in England, permitted to receive identifiable patient data for linkage.

Data privacy is a big concern and records must be linked without revealing private information about the patients. A particularly active avenue of research in record linkage is privacy-preserving record linkage (PPRL), wherein records are linked in such a way that the anonymity of the corresponding entity/individual is not compromised. These methods are extremely useful in several domains, particularly in biomedical/health and official statistics domains. Hall and Fienberg^7^ give a good overview of the PPRL problem, while^8^ present an excellent review of PPRL methods. PPRL particularly offers a great deal of utility in the context of healthcare data which is typically governed by extensive laws regarding patient privacy across countries. For example^9^, developed a PPRL approach based on a bloom filter and applied it to both simulated and real-world databases with characteristics that are similar to those found in medical databases. Another application of PPRL to health records can be found in^10^, where the authors used encryption codes and streams ciphers to link healthcare receipts to specific individuals with the stated goal of developing a larger database of healthcare insurance claims. A more recent example of the application of PPRL to healthcare records can be found in^11^; the authors use a deterministic record linkage algorithm in conjunction with hash tables to link health records across multiple sites in Chicago, Illinois. In several situations, matching variables can appear with errors and variations, and the challenge then is to link entities that are subject to error.

Two records are said to match for a corresponding entity if they match *exactly* on each element from a set of identifiers, which are referred to as *match keys*^12^. Mamun et al.^13^ proposed efficient as well as reliable sequential and parallel algorithms for linking data from different agencies. Their work is based on complete linkage hierarchical clustering algorithms for solving the record linkage problem. Another related work employed hierarchical agglomerative clustering (HAC), using single linkage^14^. Mi et al.^15^ have improved the linkage algorithms in terms of both time and space by introducing four techniques. Out of these four techniques, Faster Computation of the Edit Distance (FCED) is the most notable one. FCED predicts edit distance based on a given threshold. RLT-S is a freely available web tool for record linkage that employs a single linkage clustering algorithm^16^.

Recently, it is becoming increasingly important in many domains to address situations where the attributes of interest in record linkage are *time series* and not just individual attributes such as first and last names, street address, height, age, disease classification, etc. For instance, one could be interested in merging different time series of electro-encephalogram (EEG) records for patients, with the additional constraint that the analyst must preserve the anonymity of subjects in the databases. Since time series data exhibit dependence properties, data merging approaches for handling them must be different than those used with non-temporal records. One example can be found in^17^, in which record linkage between birth and child are used to modify time series measuring social and health outcomes for the Aboriginal/Torres Strait Islander ethnic groups. However, the authors are not linking time series but rather using record linkage of birth and health records to augment statistical time series. Another example is provided in^18^, who described an algorithm for linking records with time series.

There are also many interesting applications where the records contain time series rather than single data points or a set of uncorrelated observations. Examples are biomedical data sets with a large number of information on each patient, together with patient heart rates measured over time once every 5 min. This could be, for instance, when a patient is on a treadmill undergoing a stress test. To date, the literature on record linkage algorithms when the attributes include time series is quite sparse. In this paper, we address this problem and present a novel fast time series record linkage algorithm.

Results from our algorithm are presented in the results section. Specifically, we start by giving a detailed discussion of the performance of our algorithm for simulated experiments. We follow that with presenting and discussing our results for data obtained from *All of Us Research Program*^19,20^. Finally, we summarize and discuss our work in the discussion section.

## Results

We have carried out a comparative study of the two *TSLink* algorithms in terms of linking runtime and linking performance. The study has been evaluated twice, first with a simulated heart rate dataset in the first subsection, and next, with the real Fitbit dataset from *All of Us Research Program* in the second subsection. Since downloading the real data sets is prohibited, all our experiments have been carried out on the *AllofUs Researcher Workspace* platform in order to permit a fair comparison between simulated and real data set experiments in terms of run times. We show the setup and results from our time series record linkage algorithm.

### Results on simulated data

First, we simulate ergometer heart rate readings. We assume a sampling rate of 1 reading/second. Suppose each ergometric test is divided into 3 phases as follows:

1. Warm-up (Phase I): First 5 min 00:00–05:00 (mm:ss)
2. Stress-test (Phase II): From 05:01 to 10:00 (mm:ss)
3. Cooling-down/Recovery (Phase III): Last 5 min from 10:01 to 15:00 (mm:ss)

Each phase consequently consists of 300 readings/samples. Each ergometric test (with three phases) thus constitutes 900 points. The generated values are based on a set of random variables. These random variables define the general characteristics (the envelope) of the simulated heart rate curves, i.e., the timestamp for the first reading, the initial heart rate value, the standard deviation of noise signal and the average slope for each of the three phases.

**Figure 1:**
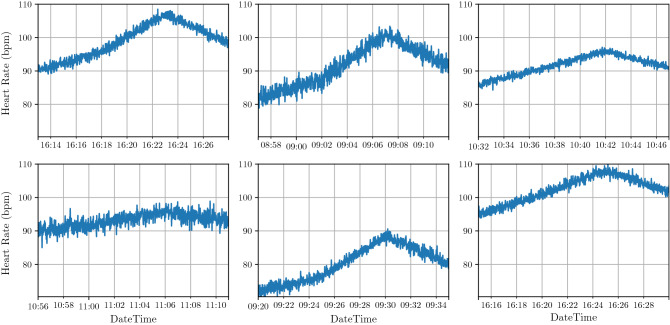
Six samples from our simulated heart rate curves.

For each patient, we simulate several heart rate ergometric test curves. For each ergometric test, we pick arbitrary values for the random variables discussed above. Then, the noise signal is added to the average slopes. This resultant composite signal becomes the simulated heart rate time series. Figure 1 shows a randomly selected sample of the simulated heart rate curves.

In our simulated data experiment, we generated a total of 32,000 ergometric tests for 1600 patients. Each patient has 20 associated tests. We saved the generated data into two separate files: ‘ergo.csv‘ and ‘pat.csv‘. The ‘ergo.csv‘ file constitutes the simulated time series data for all ergometric tests. The ‘pat.csv‘ file contains only four readings from the time series corresponding to each ergometric test. The four readings could have been picked arbitrarily. However, in our experiment we picked these readings at predefined times. Specifically, we selected the four readings corresponding to the following indices of each time series: 0, 299, 599, and 899.

**Table 1:** Linkage times in seconds for running both algorithms on the simulated data set.

Linking algorithm ergo tests	Linkage time in seconds	
	TSLink	TSLink2
4000	32.04	**1.58**
8000	139.51	**3.14**
12,000	312.20	**4.83**
16,000	557.37	**6.29**
20,000	865.19	**7.82**
24,000	1236.71	**9.39**
28,000	1688.35	**10.81**
32,000	2210.51	**12.35**

The superior linkage time in each row is highlighted in bold face.

Table 1 shows the run times of TSLink and TSLink2 algorithms on the *AllofUs Researcher Workspace* platform to link the simulated datasets. Note that our *TSLink2* algorithm achieves a speed up of up to 179. Both algorithms have $100\%$ accuracy with no false positives (FP). In the discussion section, we provide further discussion of the accuracy.

**Figure 2:**
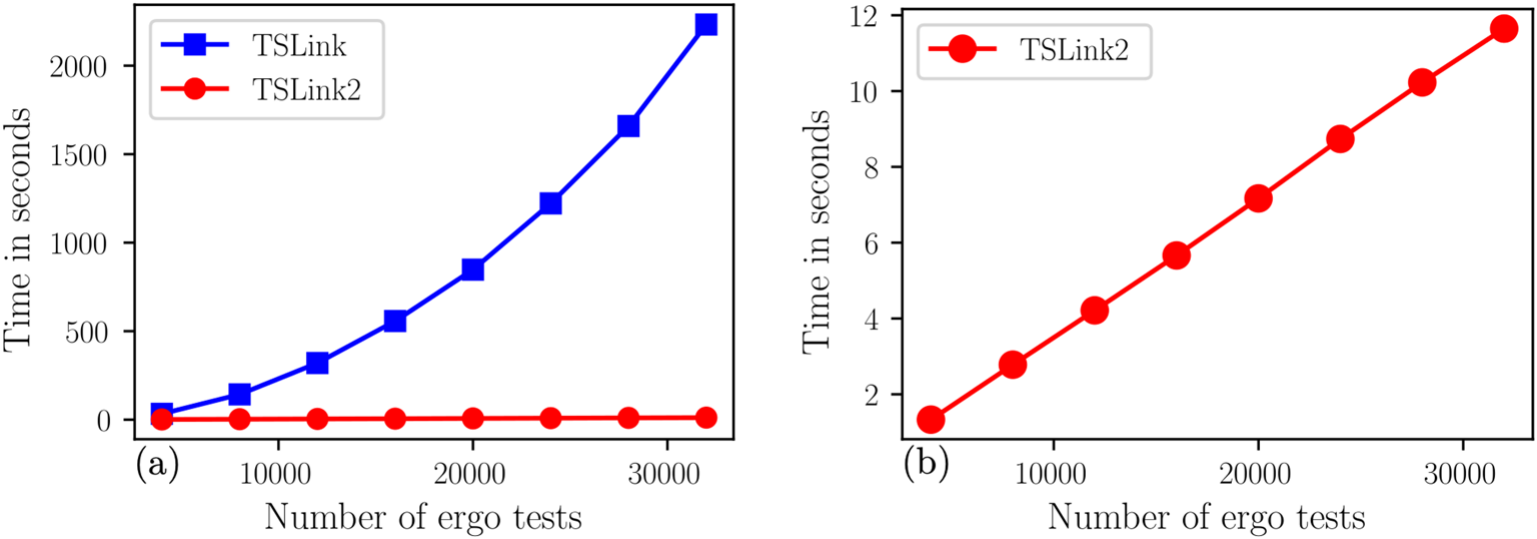
Linkage times in seconds for TSLink algorithms. (**a**) Scalability of *TSLink* algorithms on the simulated data set. (**b**) Linkage times in seconds for TSLink2 algorithm.

For a visual comparison of linkage times, please see Figure 2. In agreement with our analysis of the two presented algorithms namely, *TSLink* and *TSLink2*, Figure 2 indeed shows that *TSLink* is a quadratic time algorithm while *TSLink2* is a linear time algorithm.

### Results on real data

We conducted an experiment on real data to show that our linkage algorithm works efficiently in real scenarios. We first describe in detail the real data used in our experiment, obtained from *All of Us Research Program*^19,20^. This program allows approved researchers to use de-identified health databases in their studies on health and disease. Participants from the US voluntarily and safely share their data via this program. These shared data could be medical records, blood work, bio-samples, etc. In our study, we are particularly interested in querying the minute level heart rate table from the Fitbit dataset.

Now, we describe in detail how we used this invaluable database in our experiment. At the very beginning, we created an AllofUs Researcher Workspace for our project^21^. The minute level heart rate table of the “All of Us Registered Tier Dataset v4” is voluminous ($> 100$ Million readings). Since we only want to show a proof-of-concept and conduct a comparative study, we only worked on an arbitrarily selected subset of this Fitbit dataset. First, we filtered the minute level table to show only those participants with Fitbit data. A total of 6, 996 participants have uploaded their minute level heart rate values from their Fitbit wearable devices. We noted that some participants uploaded very few number of readings, while others generously shared a huge amount of readings. The statistical summary of the number of readings per participant is as follows: $min = 1$, $mean = 701,247.6$, $std = 574,945.2$, $max = 2,507,087$.

In order to evaluate our linkage algorithm under real scenarios, we have chosen to work on an input data that is of similar size to the previous work in^18^. The experimental data was prepared for a total of 1, 600 participants. For each participant, we requested 20 ergo tests, and required 900 readings for each test. To do this, we started by excluding all participants with number of readings $\le 18,000$. Then, we arbitrarily selected 1,600 participants. For each participant, we treated an arbitrary non-overlapping set of 900 consecutive readings as his/her set of ergo tests.

**Table 2:** Linkage times in seconds for running both algorithms on a subset of the real Fitbit dataset

Linking algorithm Ergo tests	Linkage time in seconds	
	TSLink	TSLink2
4,000	33.13	**1.33**
8,000	142.91	**2.78**
12,000	320.09	**4.21**
16,000	557.26	**5.66**
20,000	847.64	**7.17**
24,000	1221.92	**8.74**
28,000	1658.74	**10.23**
32,000	2232.38	**11.65**

The superior linkage time among each row is highlighted in bold face.

Table 2 shows the run times of TSLink and TSLink2 algorithms on the *AllofUs Researcher Workspace* platform to link the real data sets. Note that our TSLink2 algorithm achieves a speed up from about 24 up to 190. Again, both algorithms have $100\%$ accuracy with no false positives (FP).

**Figure 3:**
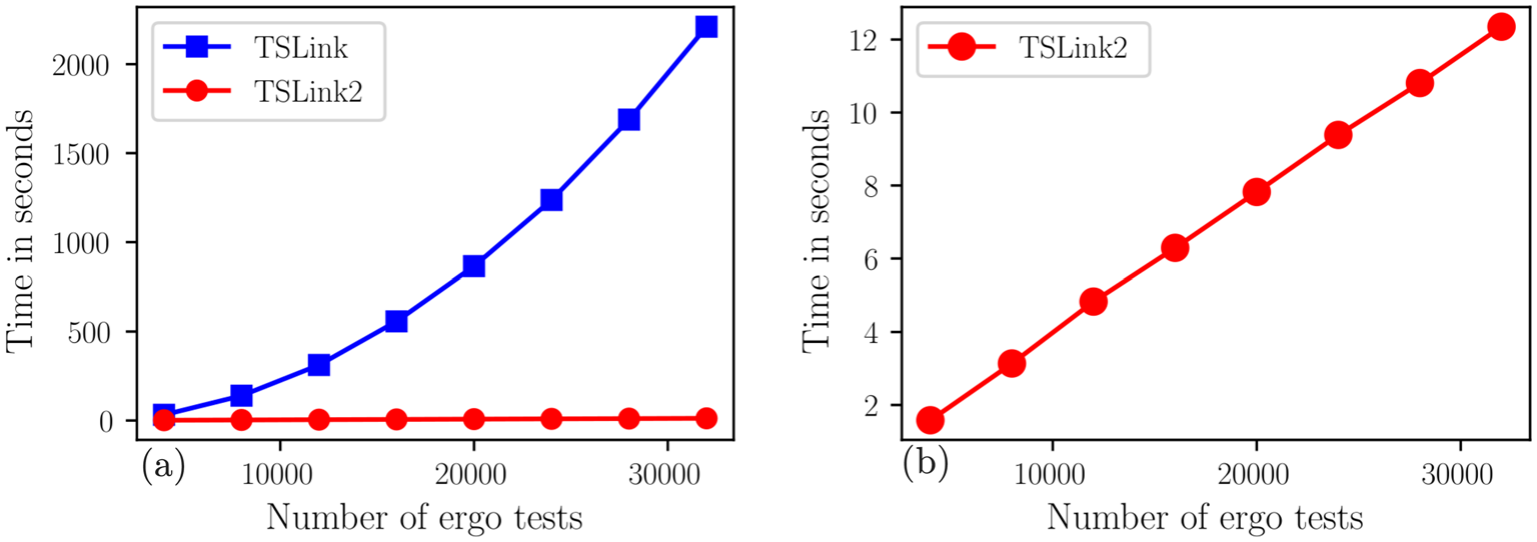
Linkage times in seconds for TSLink algorithms. (**a**) Scalability of *TSLink* algorithms on a subset of the real Fitbit dataset. (**b**) Linkage times in seconds for TSLink2 algorithm.

For a visual comparison of linkage times please see Figure 3. Similar to the experiment with simulated data, here again we see from Figure 3 that *TSLink* is a quadratic time algorithm while *TSLink2* is a linear time algorithm.

## Discussion

In this section we focus on linkage accuracy. Linkage accuracy depends on many factors. The probability or the likelihood of two or more patients/participants sharing the exact date/value pair (i.e., same minute level timestamp and same heart rate value) is one such factor. Another factor is the number of samples picked from each ergo test.

Suppose the data base contains *K* attributes on each of *n* patients. Suppose the *k*th attribute has $m_k$ possible levels, and the attributes are distributed independently of one another. For example, If heart rate is recorded to the closest integer and takes values between 60 and 220, there are 160 levels.

In record linking a database with *K* attributes, how many matches can we expect to find “by chance”? Let $p_k$ denote the probability that two randomly selected patients (out of the *n* patients) match on the *k*th attribute. Clearly,

$$\begin{aligned} p_k = 1/m_k \ \textrm{and } \ E(\textrm{match}) = \begin{pmatrix} n \\ 2 \end{pmatrix} p_k. \end{aligned}$$

Therefore, the probability that two randomly selected patients match on all *K* attributes used in the linkage algorithm is

$$\begin{aligned} p_{\textrm{all}} = \prod _{k=1}^K 1/m_k, \end{aligned}$$

and the expected number of matches is

$$\begin{aligned} E(\textrm{match}) = \begin{pmatrix} n \\ 2 \end{pmatrix} \frac{1}{\prod _{k-1}^K m_k}. \end{aligned}$$

We illustrate this in our data analysis by computing the accuracy in three different experimental settings.

In the first setting, we dropped the time attribute from the comparison. In other words, the linkage is done based on matching the date and value pairs. The TSLink algorithms then started generating false positives as expected. The results on the simulated data are as follows: *TP* $=$ 17,660 and *FP* $=$ 14,340 (i.e. $FPR = 44.81 \%$.)

In the second setting, we dropped the date attribute and kept the time. So, the linkage is done based on matching the time and value pairs. Not surprisingly, TSLink algorithms did not generate any false positives. This is because the combination of time-value pairs is quite unique across the dataset. Please note that the precision here is on the level of seconds. In other words, we have 86400 different levels for the time attribute. This high number of levels helps making the time-value pairs unique and thus the likelihood of mismatch is very low.

In the third setting, both date and time attributes are dropped. The linkage is thus based on matching the values alone. TSLink algorithms results on the simulated data are as follows: $TP = 15,461$ and $FP = 16,539$ (i.e. $FPR = 51.68\%$.)

In summary, it is clear from our results that *TSLink2* has superior performance as compared to *TSLink*. *TSLink2* is 400x faster than *TSLink* and achieves the same linkage performance. Also, it is well known that record linkage methods are generally characterized as deterministic or probabilistic methods, where the latter work by comparing two records on a number of non-unique identifiers, sometimes referred to as quasi-identifiers^22,23^. Employing the sorting idea used in *TSLINK2* in the context of a probabilistic record linkage algorithm will be interesting to investigate in the future. Another benefit from our approach is that we entirely eliminate pseudo-identifiers. Our approach uses the raw timeseries data itself.

## Methods

### Algorithm for ergometric time series

**Figure 4:**
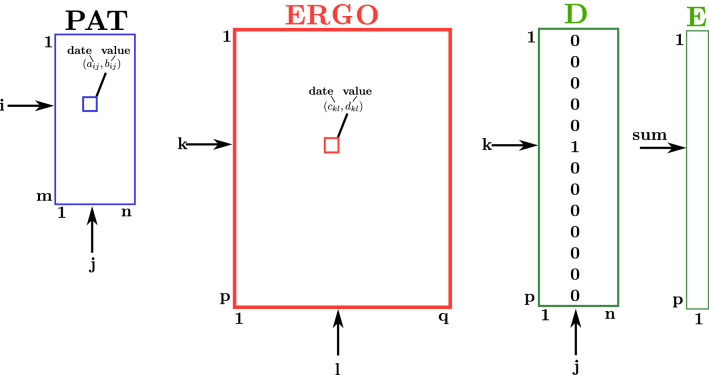
TSLink.

In this section, we give details on the algorithm of^18^. The algorithm takes as input two files: PAT and ERGO. The data in these files pertain to ergometric performance tests of patients. The number of patients was 1538. In the PAT file there are 4 (date, value) pairs for each patient. The four possible values were: start of phase 2, end of phase 2, start of phase 3, and end of phase 3. Thus we can think of the PAT file as an $N_1\times 4$ matrix where each entry in the matrix is a (date, value) pair. The ERGO file contained the performance test data. We can think of the ERGO file as an $N_2\times M_2$ matrix, where $N_2$ is the number of ergometric tests conducted and $M_2$ is the length (typically more than 4) of the time series corresponding to a test. Each row of ERGO corresponds to a performance test and any row will contain the time series corresponding to a test. Each entry in this matrix will also be a (date, value) pair. The number of performance tests conducted was 29,876. Figure 4 illustrates the matrices and pointers used in the *TSLink* algorithm.

The problem is to match the patients in PAT with performance tests in ERGO. Consider a patient *P* whose values in PAT are: $(d_1,v_1),(d_2,v_2),(d_3,v_3),$ and $(d_4,v_4)$. Let *r* be a row in ERGO. *r* will be considered as a match for this patient if these four (date, value) pairs are found in *r*. More generally, for every row *r* in ERGO, the algorithm computes the number of (date, value) pairs (from out of $(d_1,v_1),(d_2,v_2),(d_3,v_3),$ and $(d_4,v_4)$) that can be found in *r*. All the rows with the maximum number of matching (date, value) pairs will be reported as pertinent to patient *P*.

In general let the PAT matrix be of size $m\times n$ and let the ERGO matrix be of size $p\times q$. A pseudo-code for the algorithm follows: In this pseudo-code, *D* is a matrix of size $p\times n$ and *E* is an array of size $p\times 1$.
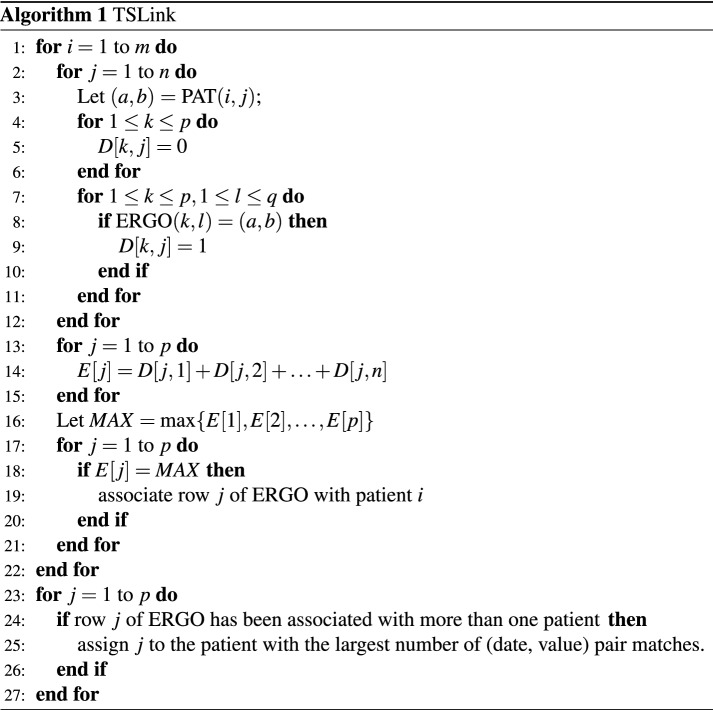


#### Analysis

The **for** loop of line 7 takes *O*(*pq*) time. The **for** loop of line 4 takes *O*(*p*) time. Thus the **for** loop of line 2 takes *O*(*npq*) time. The **for** loop of line 13 takes *O*(*np*) time. Lines 16–21 take *O*(*p*) time each. As a result, the **for** loop of line 1 takes *O*(*mnpq*) time. The **for** loop of line 23 can be completed in *O*(*mp*) time.

In summary, the run time of the algorithm *TSLink* is *O*(*mnpq*).

### A fast sorting based algorithm

In this section, we present an algorithm that is much faster than *TSLink*. The basic idea of our algorithm is to employ sorting. More details are provided below.

**Figure 5:**
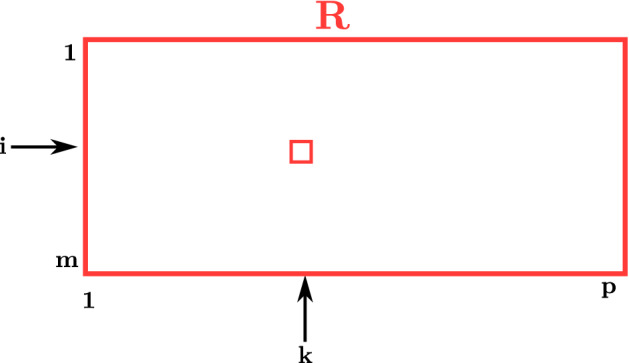
Matrix *R* used in *TSLink2*.

We denote the elements of the PAT matrix as $(a_{ij},b_{ij})$, for $1\le i\le m$ and $1\le j\le n$. Also, we denote the elements of the ERGO matrix as $(c_{kl},d_{kl})$, for $1\le k\le p$ and $1\le l\le q$. A pseudo-code for the new algorithm follows. *R* is a matrix of size $m\times p$ (see Fig. 5).
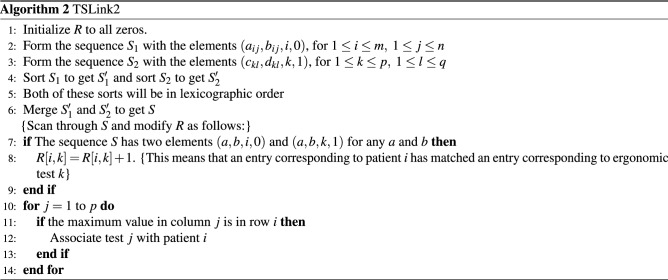


#### Analysis of algorithmic complexity

Line 1 of TSLink2 takes *O*(*mp*) time. Lines 2 and 3 take *O*(*mn*) and *O*(*pq*) times, respectively. In line 4 sorting of $S_1$ and $S_2$ will take $O(mn\log (mn))$ and $O(pq\log (pq))$ times, respectively. Merging in line 6 will take $O(mn+pq)$ time. Scanning and modifying *R* in lines 7 through 9 can be completed in $O(mn+pq)$ time. The **for** loop of line 10 takes *O*(*mp*) time. In summary, the total run time of TSLink2 is $O\left( (mn+pq)\log (mn+pq)~+~mp\right)$. Clearly, this is much better than the run time of TSLink (which is *O*(*mnpq*)).

TSLink2 is capable of linking very large number of records. Even if the size of data is too big to fit into main memory, out-of-core sorting algorithms, for example^24–28^, could be employed to sort the two sequences described above in an out-of-core fashion.

Both algorithms (TSLink and TSLink2) have been implemented using *C++11*. The source code is available on GitHub (https://github.com/ahmsoliman/tslink2).

**Figure 6:**
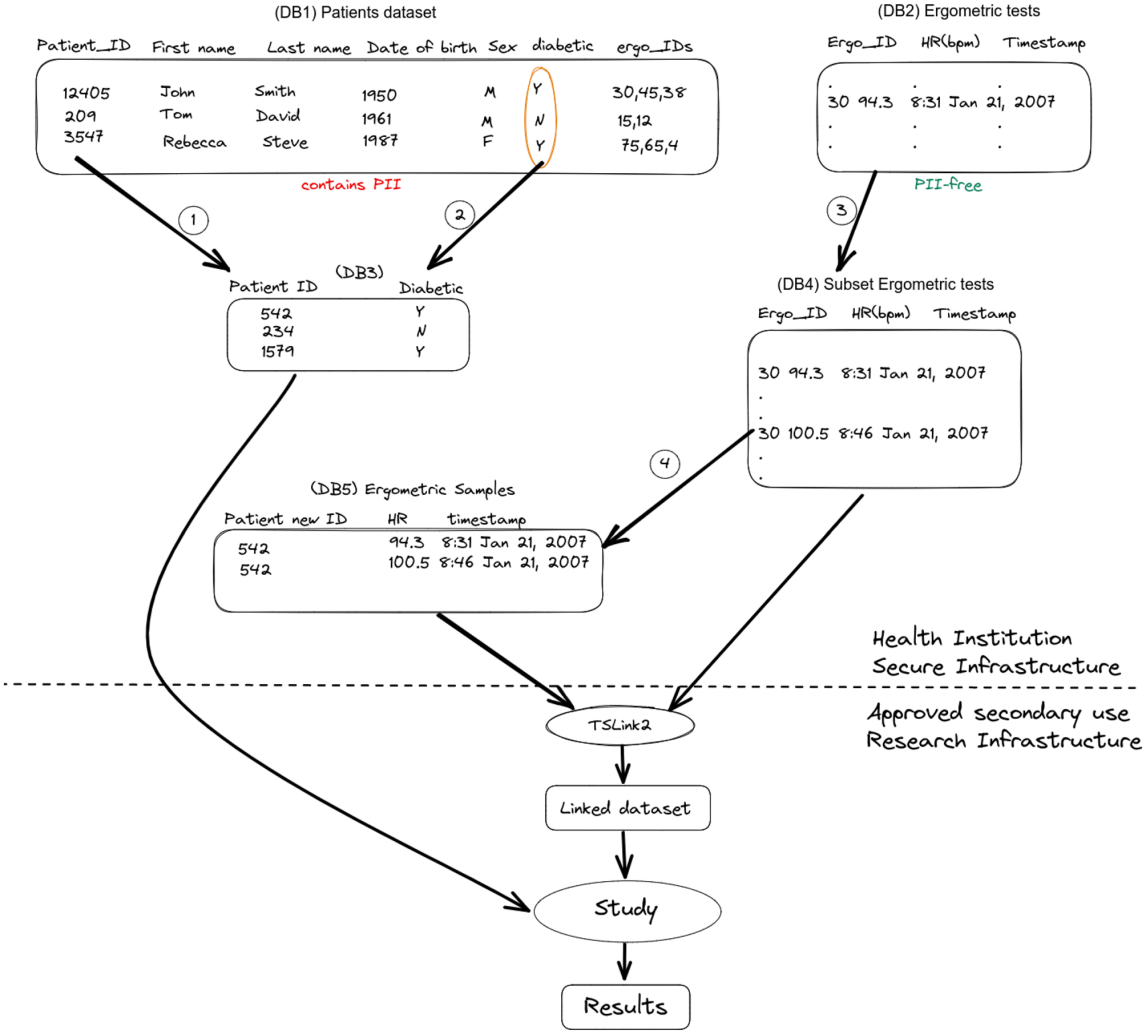
A use case for timeseries linkage: A data study secondary use workflow.

#### Application example

Imagine the following use case. A research team has been approved for conducting their secondary study on how heart rate profiles vary , if any, between diabetic and non-diabetic patients. Next, we are going to demonstrate the workflow in detail. Please see Fig. 6 and follow along. In step 1, new and unique patient identifiers are randomly generated. This step protects sensitive information in case patient IDs were based on PII information (either fully or partially) such as Social Security Numbers (SSNs) or Date of Birth (DoB). In step 2, We copy the minimal data essential for conducting the study (diabetic status in this case). In step 3, we copy all study relevant-rows from the timeseries dataset. By that we mean the rows corresponding to patients pertinent to the study. In step 4, We perform sampling from the ergometric tests. Specifically, We pick few (e.g. four) date-value pairs from each ergometric test. Then, these samples of date-value pairs are associated with the newly generated ID for the pertinent patient. Optionally, linkage is done before the dataset is released in order to verify linkage accuracy and robustness. If linkage is not robust (i.e. some false positives exist), then increase the sampling size and repeat step 4. The prepared datasets (DB4) and (DB5) are then released to the approved research team. The research team uses our *TSLink2* algorithm to link patients to their ergometric tests. Please note that this linkage employs the few date-value samples that has been released in step 4.

#### Beyond timeseries data

Despite our focus on linking timeseries data, our *TSLink2* algorithm is more generic. We have conducted two more experiments to demonstrate the versatility of our algorithm. In the first experiment, all floating point heart rate values were converted into integer values by simply dropping the fractions. Although this conversion might result in an increased probability of getting false positives, our algorithm has shown the same results (i.e. no false positives) with slightly faster linkage times.

In the second experiment, we demonstrate the ability of our algorithm to link categorical data (such as, blood type). First, we convert the heart rate values into a series of characters as follows. First the values are divided by 10, then converted into integer, and finally the integer values [0...25] are mapped into lowercase characters [ab...z]. For example, the heart rate values [94.3, 100.5, 130.1, 110.5] would be mapped into characters [*j*, *k*, *n*, *l*], respectively. Regarding the linkage time, it is even faster than the previous experiment. Regarding the linkage accuracy, as expected, the fewer number of characters creates a very high probability of sharing the same date-value pairs among different patients. Hence, 19 false positives were detected while linking 32, 000 ergo tests. Please note that, it is advised to increase the sample size to overcome this problem and reclaim the high linkage accuracy. Another solution is to utilize additional categorical data. In other words, use a group of categorical data to ensure more uniqueness across the patient records.

## Data Availability

The simulated time series data are published publicly on *figshare*^29^ and are freely available for download. Please note that due to privacy concerns, the real *Fitbit* data set is only available for researchers after joining the *AllofUs* program.
